# Roles of exosomal miRNAs from different sources in knee osteoarthritis: exercise-regulated skeletal muscle-derived exosomal miRNAs as a prospective therapeutic avenue

**DOI:** 10.3389/fmed.2026.1925853

**Published:** 2026-09-28

**Authors:** Panpan Li, Si Tang, Benxiang He

**Affiliations:** 1 School of Sports Medicine and Health, Chengdu Sport University, Chengdu, China; 2 Sichuan Academy of Chinese Medical Sciences, Chengdu, Sichuan, China

**Keywords:** exosomes, knee osteoarthritis, mesenchymal stem cells, microRNAs, muscle-derived exosomes

## Abstract

Knee osteoarthritis (KOA) is the most prevalent degenerative joint disorder in the aging population and a leading cause of joint deformity and physical disability. Long-term KOA management demands sustained pharmacological and lifestyle interventions, while advanced stages often necessitate joint replacement surgery, imposing heavy socioeconomic burdens. This narrative review examines the roles of exosomal microRNAs (miRNAs) from diverse cellular sources in KOA, with particular emphasis on exercise-regulated, skeletal muscle-derived exosomal miRNAs. A literature search of PubMed, Web of Science, and Scopus was conducted for relevant publications up to June 2026. Recent evidence demonstrates that exosomes from multiple cell types carry bioactive miRNAs that exert anti-inflammatory effects, facilitate cartilage repair, and regulate subchondral bone remodeling in KOA. While most research focuses on local joint cells and stem cell derivatives, skeletal muscle—the body’s largest endocrine organ—remains understudied. Under aging or oxidative stress, senescence-associated miRNAs such as miR-34a are upregulated in muscle-derived exosomes and delivered systemically to joint tissues, modulating chondrocyte function and skeletal stem cell activity. In contrast, exercise dynamically remodels muscle-derived exosomal miRNA profiles, driving favorable metabolic outcomes in skeletal joints via muscle–joint crosstalk. Collectively, these findings suggest that muscle-derived exosomal miRNAs represent a biologically plausible therapeutic avenue for KOA, though direct clinical validation remains to be established.

## 1 Introduction

Knee osteoarthritis (KOA) is the most common joint disease worldwide, pathologically characterized by progressive articular cartilage degeneration, osteophyte formation, subchondral bone sclerosis, and synovial inflammation and fibrosis. These pathological changes collectively result in joint dysfunction and reduced physical mobility. Current pharmacological interventions remain largely palliative, failing to halt or reverse disease progression; consequently, many patients ultimately require joint replacement surgery (1). There is, therefore, a pressing clinical need for novel therapeutic strategies capable of preventing disease progression or reversing the underlying pathological processes. In KOA, complex biomechanical changes occur within the cartilage and chondrocytes, primarily manifesting as cartilage degradation, aberrant mechanical stress, and alterations in the cartilage matrix composition. These processes are mediated by key factors such as matrix-degrading enzymes and inflammatory cytokines. Under normal joint conditions, chondrocytes reside in a relatively quiescent state with low metabolic activity. However, following articular cartilage injury, although chondrocytes initiate a repair response, the avascular nature of the cartilage matrix may cause this repair process to paradoxically exacerbate arthritis progression, resulting in a rate of cartilage matrix degradation that exceeds new matrix synthesis (2). Articular cartilage damage or inflammation accelerates joint degeneration, representing a crucial pathological link in KOA. The lack of vascular, neural, and lymphatic tissues within the joint cavity, combined with the negative-pressure microenvironment resulting from ischemia and hypoxia, poses significant challenges for cartilage repair following injury (3). Cell therapy is considered a promising interventional strategy; intra-articular injection of mesenchymal stem cells and related cell types has demonstrated potential in alleviating inflammatory responses and reducing pain (4, 5). Current research has identified exosomes as key mediators of intercellular signaling. These 30–180 nm bioactive vesicles, carrying proteins, messenger RNAs, and microRNAs, can regulate gene expression in recipient cells. They serve as important vectors for intercellular biological information exchange and are widely involved in the regulation of physiological and pathological processes. By carrying active molecules such as proteins, lipids, and nucleic acids, exosomes have been implicated in tissue repair and immune surveillance (6). The multiple biological functions of exosomes, particularly their capacity to modulate the cartilage microenvironment and promote cartilage repair, present significant application prospects in osteoarthritis treatment (7). Notably, during exercise, contracting skeletal muscles release exosomes containing specific miRNAs (8), and these exosomes act on joints, reflecting the therapeutic effects of physical activity and offering a new direction for exercise-based disease intervention. This review adopts a muscle–joint axis perspective, focusing on the potential role of skeletal muscle-derived exosomal miRNAs in exercise-mediated regulation of KOA and summarizing current evidence on exosomal miRNAs derived from different cellular sources, including mesenchymal stem cells, synovial fibroblasts, and macrophages.

## 2 Literature search strategy and methodology

A structured literature search was conducted in PubMed, Web of Science, and Scopus to identify relevant studies published up to June 2026. The search included combinations of the following terms: “exosomes,” “extracellular vesicles,” “microRNA,” “miRNA,” “knee osteoarthritis,” “cartilage repair,” “skeletal muscle,” “exercise,” “muscle-derived exosomes,” and “muscle–joint crosstalk.” The review was conducted as a structured narrative literature review, using predefined search terms and eligibility considerations to identify representative and mechanistically relevant evidence. Studies addressing the biological mechanisms, experimental evidence, or clinical relevance of exosomal miRNAs in KOA, cartilage repair, or muscle–joint communication were considered. Non-peer-reviewed preprints and conference abstracts were not considered. The identified literature was structured and narratively synthesized to compare the biological functions, potential mechanisms, and translational relevance of exosomal miRNAs from different cellular sources.

## 3 Biological functions of exosomal miRNAs in the joint disease microenvironment

### 3.1 Biogenesis and function of exosomes

Exosomes constitute a distinct subpopulation of extracellular vesicles characterized by a diameter of approximately 30–180 nm (9, 10). They are generated through the inward budding of the limiting membrane of late endosomes, producing multivesicular bodies (MVBs) containing numerous intraluminal vesicles. Upon fusion of MVBs with the plasma membrane, these intraluminal vesicles are released into the extracellular space and are subsequently designated as exosomes (11, 12). Exosomes are commonly enriched in tetraspanins such as CD63, CD9, and CD81, while the lumen harbors diverse bioactive molecules such as proteins, lipids, and nucleic acids (13, 14). Upon release into the extracellular microenvironment, exosomes deliver their cargo to recipient cells through multiple mechanisms, including ligand–receptor recognition, endocytic uptake, and direct membrane fusion, thereby mediating signal transduction and intercellular communication (15, 16).

Although exosomes were initially regarded as a cellular waste-disposal mechanism, they are now recognized as key mediators of both physiological and pathological processes (16, 17). Virtually all major cell types—including MSCs, synovial fibroblasts, macrophages, and skeletal muscle fibers—are capable of secreting exosomes, which are widely distributed across biological fluids. By transporting bioactive molecular cargo, exosomes regulate the functional status of recipient cells through paracrine or endocrine-like mechanisms and have been implicated in KOA pathogenesis and repair. Notably, muscle-derived exosomes can enter systemic circulation and exert effects on bone cells, thereby influencing skeletal homeostasis (16, 18). For example, isometric skeletal muscle contraction promotes the release of extracellular vesicles carrying relevant signaling molecules (19–21), and this muscle–skeletal signaling crosstalk may either attenuate or contribute to the progression of skeletal pathologies depending on the physiological context.

### 3.2 miRNAs: regulators of gene expression

MicroRNAs (miRNAs) are a class of non-coding small RNA molecules, approximately 22 nucleotides in length, that regulate gene expression at the post-transcriptional level (22). Their biogenesis begins with longer primary transcripts,which are sequentially processed by ribonucleases such as DROSHA and Dicer to generate mature miRNAs. These mature miRNAs are subsequently assembled into the RNA-induced silencing complex (RISC) (23, 24). These small RNAs typically function by recognizing complementary sequences within the 3'-untranslated region (3'-UTR) of target mRNAs, thereby inducing mRNA degradation or inhibiting translation (25). Studies have found that a single miRNA can regulate multiple target gene transcripts, while multiple miRNAs can also act cooperatively within the same signaling pathway,serving as important regulatory hubs for cellular function (26). In patients with KOA, 46 differentially expressed miRNAs are involved in processes such as inflammation, autophagy, chondrocyte activity,differentiation, homeostasis maintenance,metabolism, and extracellular matrix degradation (27). miRNAs in muscle have been confirmed to be key factors in the development and regulation of skeletal diseases (28). Research indicates that exosomes selectively package specific miRNAs for delivery to target cells,thereby achieving disease intervention (21, 29). Notably, one study found that skeletal muscle and other tissues can also regulate gene expression in distal tissues (skeletal diseases such as knee osteoarthritis). Therefore, exosomal miRNA transfer can be regarded as a form of endocrine-like communication. In oncology, exosomal miRNAs shuttled from tumors can reshape the microenvironment of distant tissues, contributing to disease progression (29, 30). Moreover, these miRNAs can coordinate muscle–skeletal coupling, acting as stable carriers for miRNA transport. Exosomes introduce an additional regulatory dimension to the complex muscle–skeletal crosstalk network. Specifically, skeletal muscle expresses a class of muscle-specific microRNAs (“myomiRs”, such as miR-1, miR-133a, miR-133b, and miR-206). These myomiRs promote myogenic differentiation and are released into the periphery during muscle contraction, thereby reaching distal tissues (31, 32). Notably, in the circulatory system, extracellular exosomal miRNAs exhibit remarkable stability, primarily attributed to their encapsulation within exosomes or binding to RNA-binding proteins, which protects them from RNase degradation (33). miRNAs encapsulated in exosomes not only serve as key messengers for intercellular communication but also demonstrate selective loading, meaning that specific miRNAs are preferentially sorted into exosomes and subsequently delivered to recipient cells (21, 29). This mechanism enables exosomes from tissues such as muscle to regulate gene expression in distal tissues (such as bone). Consequently, exosome-mediated miRNA transfer can be considered a form of endocrine-like communication. In parallel, within the musculoskeletal system, these miRNAs may similarly coordinate the crosstalk between muscle and bone.

### 3.3 Exosomes as mediators of intercellular crosstalk: implications for KOA

Exosomes exert their specific functions through intercellular signal transduction, thereby regulating key physiological processes such as development, cell proliferation, differentiation, and metabolism. Accumulating evidence indicates that exosomes hold significant potential in tissue regeneration, treatment of various diseases, and induction of protective mechanisms in the body (34, 35). In degenerative orthopedic diseases, exosomes have demonstrated promising biological functions, including in osteoporosis, lumbar disc degeneration, and KOA. Exosomes offer advantages such as enhanced targeting capacity, ease of storage, and convenient handling (36). Meanwhile, stem cells have garnered widespread attention in tissue regeneration research due to their dedifferentiation capacity and the beneficial components present in their secreted factors. Exosomes derived from stem cells are considered a safer next-generation alternative to cell therapy (37).

Muscle and bone communicate through multiple mechanisms, the most apparent being mechanical interaction. During contraction or exercise, skeletal muscle releases various myokines that influence bone function and metabolism, such as irisin, which promotes osteoblast differentiation and increases bone density; insulin-like growth factor 1 (IGF-1) and fibroblast growth factor (FGF-2), which participate in bone repair;and interleukin-6 (IL-6), which regulate bone metabolism (38, 39). These secreted signaling molecules establish a communication network between muscle and bone, ensuring their coordination. With the elucidation of exosome functions,studies have shown that muscle and bone exchange information through vesicle-mediated signaling (40). Exosomal miRNAs represent novel signaling molecules within this network,complementing protein-and cytokine-mediated signal transmission (41, 42). During aerobic exercise, resistance training, and high-intensity interval training, skeletal muscle may release extracellular vesicles containing miRNAs (29). Recent studies have demonstrated the existence of a directional molecular dialogue between muscle and bone, mediated by exosome-carried molecules, including microRNAs (miRNAs) (43, 44). Thus, muscle and bone together constitute an interconnected “organ system” whose regulation depends not only on mechanical forces but also on the influence of exosomal microRNAs.

## 4 Effects of stem cell-derived exosomal miRNAs on knee osteoarthritis

### 4.1 Mesenchymal stem cell-derived exosomal miRNAs

Mesenchymal stem cells (MSCs) possess broad multilineage differentiation capacity and pronounced regenerative potential. MSCs derived from diverse sources—including bone marrow, synovium, adipose tissue, peripheral blood, and umbilical cord—have demonstrated therapeutic potential in osteoarthritis (45–47). Compared with conventional cell transplantation, miRNA-enriched extracellular vesicles (EVs) have been proposed as a potential cell-free approach for KOA prevention and treatment (48, 49). A substantial body of evidence suggests that the therapeutic benefits of MSC transplantation are at least partly attributable to bioactive miRNAs carried by MSC-derived EVs. These vesicles also contain growth factors and other paracrine mediators that may collectively contribute to tissue repair, regeneration, and intercellular communication (50). Elucidating the mechanisms by which MSC-derived exosomal miRNAs from distinct sources regulate the cartilage microenvironment and contribute to cartilage repair therefore represents an important research priority.

#### 4.1.1 Bone marrow mesenchymal stem cell-derived exosomal miRNAs

Bone marrow mesenchymal stem cells (BMSCs) have emerged as one of the pivotal cell sources for regenerative therapy in knee osteoarthritis (KOA) due to their multilineage differentiation potential, immunomodulatory properties, and robust paracrine activities (51). In KOA pathogenesis, the Wnt/*β*-catenin signaling pathway represents a well-established cascade associated with bone formation, the intra-articular microenvironment, and cartilage regeneration, playing a crucial role in disease progression (52, 53). Aberrant hyperactivation of this pathway promotes the expression of catabolic genes and inflammatory mediators, thereby accelerating cartilage matrix destruction (54). BMSC-derived exosomal miRNAs have been shown to modulate this pathological cascade at specific molecular nodes, thereby promoting the maintenance of cartilage homeostasis (55). One study reported that BMSC-derived exosomal miR-127-3p directly targets and suppresses cadherin-11 (CDH11) expression, thereby blocking abnormal Wnt/β-catenin activation, suppressing IL-1β-induced inflammatory responses, and mitigating chondrocyte injury (56). Similarly, other studies have demonstrated that exosomal miR-92a-3p and miR-140-3p regulate chondrocyte matrix synthesis while suppressing chondrocyte apoptosis and degradation via the Wnt/β-catenin pathway, shifting the intra-articular microenvironment toward cartilage repair to promote cartilage regeneration (57–59).

The PI3 K/AKT/mTOR pathway is another critical intracellular cascade governing cell survival, proliferation, and metabolism in joint tissues. In KOA, dysregulated PI3 K/AKT signaling contributes to chondrocyte apoptosis, impaired autophagy, and enhanced catabolic activity, collectively accelerating cartilage degradation (60). BMSC-derived exosomal miR-206 activates the PI3 K/AKT/mTOR pathway through a KLF3-AS1/miR-206/GIT1 competitive endogenous RNA mechanism, in which lncRNA KLF3-AS1 competitively sponges miR-206 to upregulate GIT1 expression, thereby promoting chondrocyte proliferation and inhibiting apoptosis (61). Notably, BM-MSC exosomal miR-6515-5p can also inhibit the expression of the G protein-coupled receptor GRPR, thereby downregulating protein kinase B (AKT) levels, suppressing apoptosis, and protecting chondrocytes (62). Furthermore, BMSC-derived exosomal miR-326 inhibits chondrocyte apoptosis by targeting histone deacetylase 3 (HDAC3), signal transducer and activator of transcription 1 (STAT1), and nuclear factor kappa B (NF-*κ*B)/p65 signaling pathways (63). In addition, BMSC-derived exosomal miR-206 suppresses the expression of the E74-like factor 3 (Elf3) transcription factor, promoting osteoblast proliferation and differentiation, inhibiting apoptosis, alleviating inflammatory responses, and improving subchondral bone lesions in KOA (61).

More importantly, BMSC-derived exosomal miRNAs can also inhibit ferroptosis in KOA (64). Ferroptosis is an iron-dependent, non-apoptotic cell death characterized by the inactivation of the antioxidant enzyme glutathione peroxidase 4 (GPX4) and the accumulation of lipid reactive oxygen species (lipid-ROS). Recent studies have confirmed that GPX4 is a key regulator of ferroptosis (65). System Xc⁻, a cystine/glutamate antiporter, comprises SLC7A11 as its unique functional subunit; impairment of this transporter leads to intracellular glutathione (GSH) depletion, iron-dependent lipid peroxidation, and subsequent ferroptosis (66). Specifically, BMSC-derived exosomal miR-485-5p competitively sponges lncRNA SNHG7 to upregulate ferroptosis suppressor protein 1 (FSP1), thereby inhibiting IL-1β-induced chondrocyte ferroptosis and alleviating joint inflammation (67).

Furthermore, BMSC-derived exosomal miRNAs can also modulate the synovial immune microenvironment by steering macrophage polarization. Macrophages serve as the primary immune cells in the synovium, participating in inflammatory responses and tissue injury repair processes. Studies have shown that both BMSC-derived and human umbilical cord MSC-derived exosomal miRNAs possess the capacity to drive macrophage polarization from the M1 to the M2 phenotype (68, 69). One study found that upon transforming growth factor-beta 1 (TGF-*β*1) stimulation, miR-135b is highly expressed in BMSC-derived exosomes and internalized by macrophages, where it targets and inhibits mitogen-activated protein kinase 6 (MAPK6) to induce the M1 to the M2 macrophage transition, thereby reducing inflammation and facilitating cartilage damage repair (68). Further research revealed that MSCs derived from aged or inflammatory environments exhibit reduced efficacy in slowing the pathological progression of osteoarthritis (70). This underscores that despite the chondrogenic differentiation potential of MSCs, the physiological microenvironment and functional status of parent cells remain critical determinants influencing the biological activity of their exosomal miRNAs.

In summary, BMSC-derived exosomal miRNAs confer therapeutic benefit through two principal mechanisms: stable delivery of bioactive molecular cargo to target cells, and multifunctional biological activity encompassing modulation of Wnt/β-catenin and PI3 K/AKT/mTOR signaling, suppression of chondrocyte apoptosis and ECM degradation, inhibition of ferroptosis, and promotion of anti-inflammatory macrophage polarization. Collectively, these properties underscore the considerable translational potential of BMSC-derived exosomal miRNAs as therapeutic candidates for KOA.

#### 4.1.2 Adipose-derived mesenchymal stem cell exosomal miRNAs

Adipose-derived stem cells (ADSCs) and their exosomes are accessible from a readily available tissue source and have attracted considerable research interest owing to their pronounced chondrogenic differentiation potential in the treatment of articular cartilage injury. ADSCs can be obtained from multiple depots, including the infrapatellar fat pad (IPFP), inguinal adipose tissue,and subcutaneous adipose tissue. Compared with other MSC subtypes, ADSCs demonstrate relatively enhanced chondrogenic capacity and greater tolerance to inflammatory stimuli, making them an important focus of cartilage repair research (71).

ADSCs-derived exosomes miRNAs can exert anabolic effects on bone, favoring bone turnover. One study has identified miR-338-3p as an miRNA in ADSCs-derived exosomes that promotes osteoblast differentiation and bone formation. Delivery of miR-338-3p via these vesicles regulates the release of inflammatory cytokines (such as PGE2, IL-6, IL-1β, TNF-α) from chondrocytes by inhibiting RUNX2 (runt-related transcription factor2), inhibiting matrix-degrading enzymes (MMP3, MMP13), promoting chondrocyte proliferation, and suppressing apoptosis,thereby enhancing osteogenic activity (72). However, bone morphogenetic proteins (BMPs) play a critical role in knee osteoarthritis, exerting dual effects during chondrogenesis. In the early stage, BMPs promote chondrogenic differentiation,but persistent activation induces hypertrophic differentiation of chondrocytes, ultimately leading to inflammation. Cysteine-rich motor neuron protein 1 (CRIM1) is a transmembrane protein whose extracellular domain can bind to BMPs, affecting the processing, maturation, and transport of BMP precursors to the cell surface. Studies have shown that ADSC-derived exosomal miR-574-3p, upon entering cells, upregulates CRIM1 expression, inhibits BMP signaling activity, and thereby suppresses chondrocyte hypertrophy and inflammatory responses (73). Furthermore, ADSCs-derived exosomal miR-145 and miR-221 exert dual coordinated effects, with miR-221 primarily promoting cell proliferation. Sox9 and collagen type II (Col II) proteins are key proteins that promote the differentiation of periosteal cells into chondrocytes. miR-145 mainly promotes cartilage matrix formation and chondrocyte-specific gene expression by upregulating Sox9 and Col II proteins, driving periosteal cell differentiation toward the chondrocyte lineage, thereby slowing the progression of KOA (74).

ADSC-derived exosomal miRNAs also modulate joint inflammation through the regulation of autophagy. Autophagy, the process by which cells degrade and recycle intracellular components via the autophagic-lysosomal pathway, is a critical mechanism for maintaining cellular homeostasis and protecting articular cartilage (75). Exosomal miR-100-5p from IPFP-derived stem cells modulates autophagy via the mTOR pathway, and miR-429 induces autophagy by targeting FEZ2 (fasciculation and elongation protein zeta-2), collectively exerting chondroprotective effects (75, 76). Subcutaneous ADSC-derived exosomal miR-199a-3p inhibits mTOR signaling,activates autophagy,and promotes cartilage repair (77). Furthermore, ADSC-derived exosomal miR-376c-3p targets Wnt3 and Wnt9a, inhibiting the Wnt/*β*-catenin pathway and reducing cartilage matrix degradation and synovial fibrosis (78). By contrast, exosomal miR-451-5p released from tropoelastin (TE)-preconditioned ADSCs promotes ECM synthesis in chondrocytes, an effect that warrants further investigation in the context of OA progression (79). ADSC-derived exosomal miR-93-5p targets ADAMTS9, thereby suppressing inflammatory responses and attenuating cartilage damage (80).

Additional studies have found that oxidative stress can trigger inflammatory responses and matrix catabolic pathways in articular cartilage, leading to cartilage injury (81). Moreover, peroxiredoxins exert protective effects against oxidative stress-induced cartilage damage. One study demonstrated that ADSCs-derived exosomal miRNAs reduce oxidative stress and endoplasmic reticulum stress in chondrocytes, and promote the expression of the autophagy marker microtubule-associated protein 1 light chain 3 beta (MAP1LC3B), with peroxiredoxin 6 (Prdx6) and miR-486-5p playing key roles (82–84).

#### 4.1.3 Synovial mesenchymal stem cell-derived exosomal miRNA

Synovial mesenchymal stem cells (SMSCs) have been demonstrated to possess multilineage differentiation potential and have been applied in animal model experiments of knee osteoarthritis intervention (84). However, the specific mechanisms remain to be further elucidated. In one study, miR-302c was identified as an miRNA in SMSCs-derived exosomes. The findings revealed that this miRNA can target and regulate a disintegrin and metalloproteinase 19 (ADAM19), promote chondrocyte proliferation, inhibit cartilage inflammation and extracellular matrix (ECM) degradation, thereby facilitating cartilage repair in osteoarthritis (85). Notably, Runx2 (runt-related transcription factor 2) expression is elevated in the synovium of KOA, where it directly activates matrix metalloproteinase 13 and induces chondrocyte hypertrophy, thereby directly leading to cartilage matrix destruction. Synovial mesenchymal stem cell-derived exosomal miR-155-5p promotes chondrocyte proliferation, migration, ECM secretion, and inhibits apoptosis by targeting and suppressing Runx2, thus promoting cartilage repair (86). Neuropilin-1 (NRP-1) is a transmembrane protein widely expressed in various tissues and has recently been implicated in the regulation of bone metabolism. Studies have shown that SMSC-Exos upregulate miR-485-3p, which inhibits NRP-1 expression, leading to inactivation of the PI3 K/Akt signaling pathway, thereby promoting chondrocyte proliferation and migration, reducing inflammatory responses, and suppressing ECM degradation (87, 88).

SMSCs-derived exosomal miRNAs have also been shown to alleviate inflammatory responses through regulation of macrophage polarization. Specifically, in KOA, ANXA2 (annexin A2) expression is elevated, promoting activation of the TLR4/MyD88/NF-*κ*B pathway while inhibiting M2-type polarization, leading to increased inflammation levels in KOA. SMSC-Exos carrying miR-3614-5p are delivered via extracellular vesicles to annexin A2 (ANXA2), inhibiting the pro-inflammatory TLR4/MyD88/NF-*κ*B pathway, thereby promoting macrophage polarization from the M1 to the M2 phenotype and alleviating inflammation and cartilage damage (89).

In experiments involving IL-1β-induced chondrocytes,both miR-129-5p and miR-4738-3p derived from SMSCs exosomes were shown to alleviate IL-1β-induced chondrocyte inflammation by targeting and inhibiting the TLR4/NF-*κ*B pathway (90, 91). Notably, SMSC-Exos are enriched in miR-140-5p and miR-212-5p, both of which downregulate various inflammatory mediators (including interleukin-6, C-C motif chemokine ligand 2, tumor necrosis factor-alpha, cyclooxygenase-2, and inducible nitric oxide synthase) by targeting and inhibiting E74-like Ets transcription factor 3 (ELF3) expression. Simultaneously, miR-140-5p enhances chondrocyte proliferation and migration capabilities without disrupting cartilage matrix integrity, thereby representing a candidate therapeutic approach (92, 93).

#### 4.1.4 Umbilical cord mesenchymal stem cell-derived exosomal miRNAs

Clinically, umbilical cord mesenchymal stem cells (HucMSCs) have shown great potential in the treatment of KOA due to their regenerative and anti-inflammatory effects (94–96). However, among various types of MSCs, umbilical cord-derived MSCs (UCMSCs) have received particular attention due to their angiogenic and osteogenic properties, as well as their non-invasive accessibility and strong regenerative capacity (97, 98). NOX4 (NADPH oxidase 4) is a member of the NADPH oxidase family. In chondrocytes, NOX4 overexpression induces oxidative stress, damages intracellular proteins, lipids, and DNA, and ultimately leads to cell apoptosis. Furthermore,extracellular vesicle-derived miR-100-5p and miR-23a-3p delivered by umbilical cord mesenchymal stem cells inhibit the expression of NOX4 and phosphatase and tensin homolog (PTEN), upregulate protein kinase B (AKT) levels, alleviate oxidative stress and apoptosis, and promote cartilage regeneration (69, 99). In the skeletal joint, the pro-inflammatory molecule METTL3 (methyltransferase-like protein 3) increases the expression of NLRP3 (NOD-like receptor family pyrin domain containing 3) in the chondrocyte extracellular matrix, ultimately leading to elevated inflammation levels. HucMSC-EVs, via miR-1208, target METTL3 to reduce the m6A level of NLRP3 mRNA, inhibit the secretion of pro-inflammatory factors and the degradation of cartilage ECM, thereby alleviating the progression of KOA (100). In terms of cartilage repair, umbilical cord-derived MSCs and MSCs differentiated from pluripotent stem cells have demonstrated comparatively greater chondrogenic effects than adipose-derived MSCs in certain contexts. Notably, a previous study utilized dual-engineered targeting to continuously release umbilical cord-derived mesenchymal stem cell exosomal miRNAs targeting chondrocytes, promoting hyaline cartilage regeneration and achieving joint structural repair and functional recovery (101). In another related study, it was found that umbilical cord mesenchymal stem cell exosomal miR-223 underwent surface CTP targeting and electroporation dual-engineering treatment. miR-223 regulates NLRP3 inflammasome-mediated pyroptosis in chondrocytes, alleviating joint inflammation (102). Furthermore,in advanced KOA, bone and joint pain represents a typical neuropathic pain,with central sensitization being an important mechanism underlying the persistence and exacerbation of OA pain. Long non-coding RNAs (LncRNAs) can act as molecular sponges for specific miRNAs, thereby inhibiting the latter’s regulatory effects on their target genes. For example, lncRNA H19 in HucMSCs-derived exosomes relieves advanced arthritis pain by adsorbing miR-29b-3p, which in turn inhibits the central sensitization protein FOS.Moreover, miR-29b-3p upregulates Smad1 and Smad3 levels in the transforming growth factor-β signaling pathway and inhibits the expression of Forkhead box O3 (FOXO3), thereby promoting cartilage repair and regeneration (103). As research into mesenchymal stem cell biology continues to advance, the therapeutic potential of additional MSC subtypes warrants systematic investigation in future studies.

### 4.2 Muscle-derived exosomal miRNAs

#### 4.2.1 Advantages of muscle-derived exosomes and the modulatory effects of exercise variables on exosomal miRNAs

Compared with exosomes derived from other cellular sources—such as mesenchymal stem cells or chondrocytes—skeletal muscle-derived exosomes possess distinctive biological and practical advantages. From a biological standpoint, skeletal muscle constitutes the largest endocrine organ in the human body and generates substantial quantities of exosomes both at rest and in response to contractile activity; during exercise, muscle-derived extracellular vesicles have been estimated to account for approximately 1%–5% of the total circulating extracellular vesicle pool (104). Unlike exosomes derived from joint-local sources such as synovial fibroblasts or chondrocytes, muscle-derived exosomes are released into the systemic circulation and can therefore exert regulatory effects on anatomically distant tissues—including articular cartilage and subchondral bone—without necessitating intra-articular delivery. From a practical standpoint, the miRNA cargo of skeletal muscle-derived exosomes is amenable to modulation through physical exercise or resistance training; exercise thus functions as a form of *in vivo* exosomal miRNA remodeling, reshaping the miRNA profile without requiring *ex vivo* modification or synthetic delivery vectors (105).

Current evidence indicates that exercise type, intensity, duration, and frequency differentially modulate the miRNA cargo of muscle-derived exosomes (106). With respect to exercise modality, continuous aerobic training (CAT) and high-intensity interval training (HIIT) elicit distinct exosomal miRNA signatures in humans: although both modalities modulate pathways involved in autophagy and cell fate regulation, their downstream target gene networks diverge—CAT-associated miRNAs being preferentially enriched in cognitive function and substrate metabolism, whereas HIIT-associated miRNAs are enriched in organ growth and cardiac muscle function (104). Specifically, HIIT has been reported to induce a 2- to 3-fold increase in circulating exosomal miR-133a (107). It should be noted that while miR-133a is also elevated under conditions of skeletal muscle atrophy as a marker of pathological muscle wasting, exercise-induced upregulation of circulating exosomal miR-133a reflects an adaptive metabolic response (32), underscoring the context-dependency of myomiR function. Resistance exercise training (RET) further remodels the exosomal miRNA landscape by upregulating circulating exosomal miR-486-5p and miR-215-5p in trained individuals (108). Exercise duration likewise influences muscle-derived exosomal miRNA composition: acute exercise and long-term training alter exosomal miRNA profiles through distinct mechanisms—acute bouts rapidly mobilize pre-formed exosomal cargo into systemic circulation, whereas long-term training progressively remodels the baseline exosomal secretion profile of skeletal muscle (104, 109). Notably, whether low-intensity exercise is sufficient to meaningfully alter muscle-derived exosomal miRNA cargo remains an unresolved question and represents an important gap in current knowledge.

In older individuals, the levels of canonical myomiRs—including miR-1, miR-133a, miR-133b, and miR-206—in muscle-derived exosomes are significantly attenuated relative to younger counterparts (18), whereas senescence-associated miR-34a is markedly upregulated in aged muscle-derived exosomes (110). This age-dependent shift in exosomal miRNA cargo impairs osteoprogenitor cell function and contributes to KOA progression. Importantly, RET has been shown to partially restore the expression of circulating exosomal myomiRs in older individuals (104), and exercise-mediated attenuation of miR-34a release may similarly reduce osteoprogenitor senescence and preserve osteogenic capacity. Collectively, these findings suggest that exercise may counteract the age-related deterioration of the muscle–exosome signaling axis, providing a mechanistic rationale for exercise-based intervention in older patients with KOA.

It is worth noting that although exosomes derived from MSCs, ADSCs, and other cellular sources have accumulated substantial preclinical evidence in cartilage repair, their clinical translation remains constrained by inherent limitations, including difficulties in *ex vivo* production standardization, immunogenic risks associated with allogeneic origin, and dependence on intra-articular injection for targeted delivery (32, 107). By contrast, autologous muscle-derived exosomes induced through exercise carry a lower risk of immune rejection, and exercise can actively remodel their miRNA profile from a pro-inflammatory and pro-senescent state toward an anti-inflammatory and anti-aging phenotype, enabling active modulation of the KOA joint microenvironment via targeting of key signaling pathways including NF-*κ*B, Wnt/β-catenin, and PI3 K/AKT (108, 109). This exercise-mediated dynamic remodeling capacity represents a physiological property not readily replicated by exosomes from other cellular sources. It should be noted, however, that direct head-to-head comparative evidence with exosomes from other cellular sources is currently lacking, and the clinical translational value of these advantages warrants further validation through systematic investigation.

Beyond their therapeutic implications, circulating muscle-derived exosomal miRNAs hold promise as non-invasive biomarkers for KOA monitoring. Muscle-enriched miRNAs such as miR-206 and miR-133a are detectable in peripheral blood and have been proposed as candidate biomarkers of musculoskeletal status and aerobic exercise capacity (18). However, a critical methodological caveat must be acknowledged: given that muscle-derived extracellular vesicles constitute only a minor fraction of the total circulating vesicle pool, and that no tissue-exclusive exosomal surface markers have yet been validated, the precise attribution of circulating miRNA changes to skeletal muscle origin remains challenging (104). Standardized isolation protocols, muscle-specific extracellular vesicle tracking tools, and adequately powered prospective studies are therefore prerequisites for the clinical translation of these biomarker candidates.

#### 4.2.2 Effects of muscle-derived exosomal miRNAs on KOA

Exosomal miRNA-mediated muscle-to-bone signaling can exert both beneficial and detrimental effects on KOA, depending on the prevailing physiological context. Skeletal muscle atrophy promotes the release of muscle-derived exosomal miRNAs that negatively impact bone formation. Notably, miR-34a has been identified as a miRNA that is upregulated in aged muscle-derived exosomes; circulating muscle-derived miR-34a has been demonstrated to downregulate Sirtuin 1 (Sirt1) and runt-related transcription factor 2 (Runx2), driving osteoprogenitors toward a senescent, non-osteogenic phenotype and ultimately inducing senescence in bone marrow mesenchymal stem cells (MSCs) (111). Given that BMSC-derived exosomal miRNAs have been shown to regulate KOA-associated inflammation and promote cartilage regeneration (51, 56, 63), atrophy-derived miR-34a impairs bone marrow stem cell function and thereby establishes a pathological link between skeletal muscle atrophy and KOA progression. In addition to miR-34a, other muscle-derived miRNAs can also influence the development of skeletal diseases including KOA; for example, skeletal muscle atrophy is associated with elevated circulating exosomal levels of miR-133a and miR-206, which may in turn affect cartilage remodeling (18, 110).

Conversely, under conditions of physical exercise or mechanical loading, miRNAs within muscle-derived exosomes can exert anabolic effects on bone. Evidence indicates that exercise remodels the miRNA composition of muscle-derived exosomes toward a profile more conducive to skeletal metabolic homeostasis (104, 106). Impaired osteoblast function has been shown to exacerbate KOA progression, whereas restoration of osteoblast activity attenuates disease progression (111). miR-27a-3p was identified as a muscle-derived exosomal miRNA that promotes osteoblast differentiation. Osteoblasts internalize myogenic exosomes, which deliver miR-27a-3p to directly target and downregulate adenomatous polyposis coli (APC), thereby activating the Wnt/β-catenin pathway and enhancing osteogenic activity (105, 112). Collectively, these findings suggest that muscle-derived exosomal miRNAs can function analogously to “exerkines,” exerting positive regulatory effects on muscle–skeletal crosstalk during exercise-induced processes.

In summary, skeletal muscle communicates with bone not only through mechanical forces and secreted proteins but also via exosomal miRNAs; depending on the prevailing physiological context—such as aging or exercise—these miRNAs may either retard or promote KOA progression. This bidirectional crosstalk underscores the therapeutic potential of targeting muscle-derived exosomal miRNAs to modulate subchondral bone remodeling and cartilage homeostasis. It should be noted that muscle-derived extracellular vesicles constitute only a minor fraction of the total circulating extracellular vesicle pool, and future investigations should prioritize elucidating the cargo specificity, delivery efficiency, and mechanism of action of muscle-derived exosomal miRNAs, thereby laying the groundwork for exercise-based precision interventions in KOA, as illustrated in Figure 1 and summarized in Table 1.

**Figure 1:**
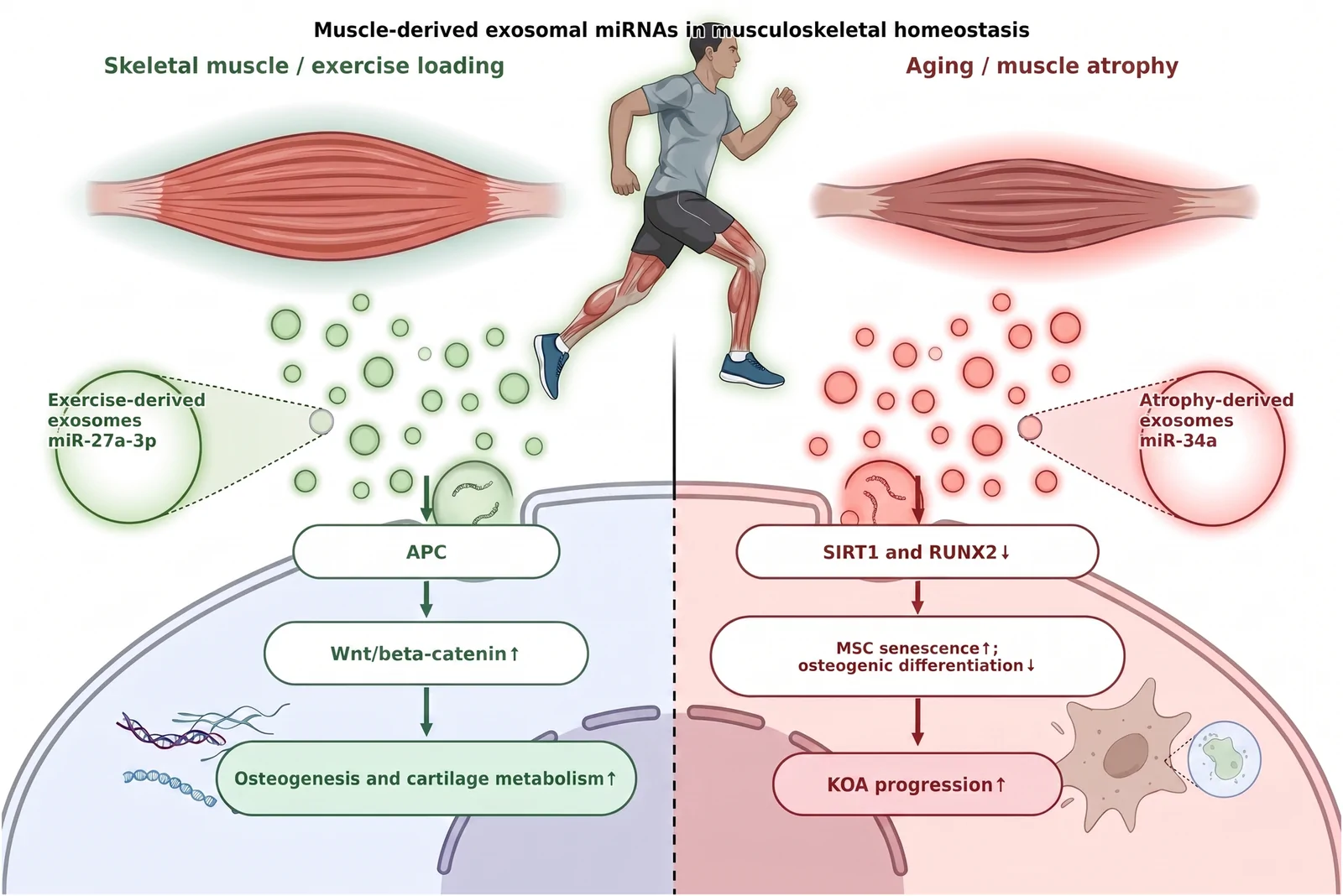
Exercise-induced remodeling of the skeletal muscle-derived exosomal miRNA profile and its role in muscle-to-bone communication. Exercise upregulates miR-27a-3p in muscle-derived exosomes, activating Wnt/β-catenin signaling in osteoblasts to promote bone formation, while concurrently attenuating miR-34a release to reduce osteoprogenitor senescence. These coordinated changes establish a positive feedback loop reinforcing both skeletal muscle and bone health.

**Table 1:** Mechanisms of action of exosomal miRNAs from different cellular sources in the cartilage repair process of osteoarthritis.

Cell Source	Exosomal miRNA	Target/Signaling Pathway	Mechanism of Action in KOA
BMSCs	miR-206	KLF3	Promotes osteoblast proliferation and differentiation in KOA by suppressing Elf3 expression.
	miR-127-3p miR-140-3p	Wnt/*β*-catenin	Regulate chondrocyte matrix synthesis and suppress apoptosis and ECM degradation via the Wnt/*β*-catenin pathway, promoting cartilage regeneration.
	miR-6515-5p	AKT	Downregulates AKT activity, suppresses chondrocyte apoptosis, and exerts chondroprotective effects.
	miR-326	HDAC3/STAT1/NF-*κ*B	Inhibits chondrocyte apoptosis by targeting HDAC3 and modulating STAT1 signaling.
	miR-485-5p	lncRNA SNHG7/FSP1	Competitively sponges lncRNA SNHG7 to upregulate FSP1, inhibiting IL-1β-induced chondrocyte ferroptosis and alleviating joint inflammation.
	miR-135b	MAPK6	Drives M1-to-M2 macrophage polarization by targeting MAPK6, reducing inflammation and promoting cartilage repair.
ADSCs	miR-338-3p	RUNX2	Promotes chondrocyte proliferation and inhibits apoptosis by suppressing RUNX2, reducing pro-inflammatory cytokines and matrix-degrading enzymes.
	miR-574-3p	CRIM1/BMPs	Upregulates CRIM1 to inhibit BMP signaling, suppressing chondrocyte hypertrophy and inflammatory responses.
	miR-145 miR-221	Sox9, Col II	Promotes the chondrogenic differentiation of periosteal cells, thereby delaying the progression of KOA
	miR-100-5p	mTOR	Modulates autophagy via the mTOR pathway, exerting cartilage-protective and reparative effects.
	miR-429	FEZ2	Induces autophagy by targeting FEZ2, contributing to cartilage protection and repair.
	miR-199a-3p	mTOR	Inhibits mTOR signaling, activates autophagy, and promotes cartilage repair.
	miR-376c-3p	Wnt3, Wnt9a	Targets Wnt3 and Wnt9a to inhibit Wnt/β-catenin pathway, reducing cartilage matrix degradation and synovial fibrosis.
	miR-93-5p	ADAMTS9	Suppresses inflammatory responses and mitigates cartilage damage by targeting ADAMTS9.
	miR-486-5p	Prdx6	Reduces oxidative stress responses in chondrocytes.
SMSCs	miR-155-5p	Runx2	Promotes chondrocyte proliferation, migration, and ECM secretion while inhibiting apoptosis by suppressing Runx2.
	miR-302c miR-129-5p	ADAM19	Inhibit extracellular matrix degradation and inflammation
	miR-485-3p	NRP-1	Enhances chondrocyte proliferation and migration while reducing inflammation and preventing ECM breakdown.
	miR-4738-3p miR-3614-5p	ANXA2	Promotes M1-to-M2 macrophage polarization by targeting ANXA2 to inhibit TLR4/NF-κB signaling, alleviating inflammation and cartilage damage.
	miR-140-5p miR-212-5p	ELF3	Downregulate inflammatory mediators (IL-6, CCL2, TNF-α, COX-2, iNOS) by targeting ELF3.
HucMSCs	miR-100-5p miR-23a-3p	NOX4/PTEN/AKT	Inhibit NOX4, alleviate oxidative stress and apoptosis, and promote cartilage regeneration.
	miR-1208	METTL3	Targets METTL3 to reduce, inhibiting pro-inflammatory cytokine secretion and protecting against cartilage ECM degradation.
	miR-223	NLRP3	Regulates NLRP3 inflammasome-mediated pyroptosis in chondrocytes, alleviating joint inflammation.
	miR-29b-3p	Smad1, Smad3	Upregulates Smad1 and Smad3, promoting cartilage repair and regeneration.
Muscle-derived	miR-34a	Sirt1, Runx2	Reduces Sirt1 and Runx2 expression, contributing to KOA disease progression
	miR-27a-3p	APC	Activate the Wnt/β-catenin signaling pathway, thereby enhancing osteogenic activity.
Synovial fibroblast-derived	miR-106b	lncRNA H19	Reverses cartilage injury and drives cartilage repair by blocking matrix degradation and boosting chondrocyte proliferation and migration
	miR-19b-3p	SLC7A11	Inhibits SLC7A11, exacerbating chondrocyte ferroptosis and cartilage injury.
	miR-214-3p miR-182-5p miR-126-3p	—	Inhibit osteophyte formation and prevent cartilage degeneration, exerting anti-inflammatory and anti-apoptotic effects to protect articular cartilage.
Macrophage-derived	miR-26b-5p	TLR3	Drives M1-to-M2 macrophage polarization by targeting TLR3 alleviating inflammation and cartilage degeneration.
	miR-124-3p	STAT3	Inhibits STAT3 phosphorylation under hypoxic conditions, promoting chondrocyte proliferation and inhibiting apoptosis and ECM degradation.
Osteoclast-derived	miR-212-3p	TGF-β1/Smad2	Promotes cartilage matrix degradation.
Platelet-rich plasma-derived	miR-25-3p	MAP4K2	Targets MAP4K2 to suppress Hippo pathway activation, activating TNFAIP3 and effectively inhibiting chondrocyte inflammatory responses.
Human urine-derived stem cell	miR-140-5p	VEGFA	Inhibits oxidative stress responses in chondrocytes, reduces apoptosis, improves subchondral bone remodeling, and exerts protective effects on articular cartilage.
Mammalian and plant-derived	miR-143 miR-124	ROCK1	Suppress pro-inflammatory regulators ROCK1 via promoter methylation, reducing cartilage degradation and alleviating KOA inflammation.
	miR-146b-5p	TRAF6	Inhibit extracellular matrix degradation, protect chondrocytes, and promote chondrocyte repair.
	miR-140	MMP13	Targets MMP13 to suppress cartilage matrix degradation, exerting dual chondroprotective and anti-inflammatory effects.

KOA, knee osteoarthritis; BMSCs, bone marrow mesenchymal stem cells; ADSCs, adipose-derived stem cells; SMSCs, synovial mesenchymal stem cells; HucMSCs, human umbilical cord mesenchymal stem cells; IPFP, infrapatellar fat pad; EV, extracellular vesicle; PRP, platelet-rich plasma; OA, osteoarthritis; MSC, mesenchymal stem cell.

### 4.3 Synovial fibroblast-derived exosomal miRNAs

In osteoarthritis, the pathological changes primarily include bone formation, cartilage degeneration, and alterations in synovial structure and cells. Synoviocytes primarily comprise macrophage-like synoviocytes and fibroblast-like synoviocytes. Notably, activated fibroblast-like synoviocytes have been identified as key drivers of osteoarthritis, secreting humoral mediators that sustain inflammatory processes, proteases that lead to cartilage and bone destruction, and factors that drive fibrosis, thereby attracting increasing attention (113, 114). Exosomal miRNAs derived from synovial fibroblasts exert differential effects on chondrocytes. For instance, miR-19b-3p, miR-106b, and miR-142-5p exacerbate articular cartilage injury, whereas miR-214-3p, miR-182-5p, and miR-126-3p have the potential to promote articular cartilage repair (64, 115–119). In noteworthy studies, miR-106b carried by synovial fibroblast-derived exosomes was found to target pyruvate dehydrogenase kinase 4 (PDK4) and regulate the RANKL/RANK/OPG signaling pathway, thereby promoting cartilage injury. This study suggests that miR-106b represents a potential therapeutic target. Furthermore, the interaction between miR-106b and long non-coding RNA H19 through competitive binding to miR-106b provides a promising approach for reversing cartilage injury and promoting cartilage repair (115). Ferroptosis, a form of cell death characterized by disruption of redox homeostasis, mitochondrial dysfunction, and iron-dependent metabolic dysregulation, plays an important role in regulating cartilage inflammatory responses. Studies have shown that miR-19b-3p carried by fibroblast-like synoviocyte-derived exosomes is delivered via extracellular vesicles and inhibits solute carrier family 7 member 11 (SLC7A11), thereby exacerbating chondrocyte ferroptosis and cartilage injury (64, 113). Collectively, the detrimental effects of synovial fibroblasts on cartilage repair pathways reveal the destructive role of inflammation in cartilage integrity, emphasizing that cartilage repair strategies must incorporate inflammation inhibition as a key component.

### 4.4 Macrophage-derived exosomal miRNAs

Macrophages, as one of the immune cell types, can polarize into M1 (pro-inflammatory) and M2 (anti-inflammatory) phenotypes in KOA. Following polarization, macrophages regulate chondrocyte function predominantly in a paracrine manner by secreting a multitude of inflammatory cytokines, matrix metalloproteinases, and growth factors, thereby modulating chondrocyte metabolism and contributing to joint pain and cartilage inflammation. Therefore, macrophages play a crucial role in regulating bone metabolism and maintaining intra-articular homeostasis (120, 121). Studies have shown that M1 macrophage-derived exosomal miR-363 upregulates the expression of interleukin-6 (IL-6) and tumor necrosis factor-alpha (TNF-α) in chondrocytes by inhibiting GTPase-activating protein-binding protein2 (G3BP2), thereby exacerbating cartilage injury (121). These findings elucidate the mechanism by which M1 macrophage exosomal miRNAs aggravate cartilage damage. Furthermore, M2 macrophages exert anti-inflammatory effects in KOA. In a noteworthy study, M2 macrophage-derived exosomal miR-26b-5p exerts a dual effect. First, it regulates macrophage polarization toward the M2 anti-inflammatory phenotype by targeting Toll-like receptor 3 (TLR3), thereby blocking NF-*κ*B-mediated inflammatory responses. Second, it downregulates collagen type X alpha 1 chain (COL10A1) and suppresses chondrocyte hypertrophy, thereby alleviating inflammation and cartilage degeneration in KOA (122). Notably, M2 macrophage-derived exosomal miR-124-3p under hypoxic conditions exerts a protective effect against anterior cruciate ligament transection-induced KOA. Specifically, hypoxic M2 macrophage-derived exosomal miR-124-3p inhibits the phosphorylation of signal transducer and activator of transcription 3 (STAT3), thereby promoting chondrocyte proliferation and inhibiting apoptosis and ECM degradation (123). Therefore, stimulating macrophage polarization from the pro-inflammatory M1 to the anti-inflammatory M2 phenotype using exosome-carried miRNAs may represent a promising therapeutic strategy for KOA.

### 4.5 Mammalian and plant-derived exosomal miRNAs

Mammalian and plant-derived exosomes are extracellular vesicles secreted by cells, containing abundant bioactive substances such as proteins,nucleic acids, and lipids (124, 125). Recently, there has been a surge in reports on the use of mammalian and plant-derived exosomes for the treatment of skeletal and joint diseases. Studies have shown that bioactive compounds extracted from certain herbs contain active ingredients with anti-inflammatory, anti-apoptotic, and anti-oxidative stress effects (126). Methods for isolating bioactive substance-enriched vesicles from herbs have been established, providing a new direction for the future application of traditional Chinese medicine in the treatment of skeletal and joint diseases (127). Curcumin-induced modulation of miRNA promoter methylation upregulates the expression of miR-143 and miR-124, thereby regulating pro-inflammatory factors such as Rho-associated coiled-coil-containing protein kinase 1 (ROCK1) and nuclear factor kappa-B (NF-*κ*B), reducing cartilage degradation, alleviating inflammation, and promoting cartilage repair in KOA (128). In a noteworthy study, exosomal miR-146b-5p treated with fucoidan inhibited TNF receptor-associated factor 6 (TRAF6), subsequently blocking the PI3 K/AKT/mTOR pathway, thereby reducing joint inflammation and ECM degradation, protecting chondrocytes, and promoting chondrocyte repair (129). Furthermore, one study found that antler stem cell-derived exosomal miR-140, isolated from the unossified antlers of male sika deer, exerted dual effects encompassing cartilage repair and inflammation inhibition in a KOA model by targeting MMP13 to suppress cartilage matrix degradation and NLRP3 inflammasome activation. This study not only revealed the molecular basis of the regenerative potential of antler organs but also provides a potential direction for cell-free therapy in KOA (130). In summary, the above studies indicate that miRNAs carried by extracellular vesicles derived from mammals and plants offer a promising approach for the treatment of KOA.

### 4.6 Exosomal miRNAs from other cell types

Exosomal miRNAs derived from other cell types may also exert positive effects on KOA. Studies have shown that exosomal miRNAs from osteoclasts, human urine-derived stem cells, and platelets demonstrate therapeutic promise for KOA (131–133). Specifically, one study confirmed that osteoclast-derived exosomal miR-212-3p promotes cartilage matrix degradation by regulating the transforming growth factor-*β*1/Smad family member 2 signaling pathway. This finding suggests that inhibiting miR-212-3p expression may facilitate cartilage repair and supports the TGF-*β*1/Smad2 axis as a potential target for promoting cartilage repair (133). Additionally, osteoclast-derived exosomal miRNAs such as miR-21a-5p, miR-214-3p, miR-30a-5p, and miR-30d-5p target and inhibit the expression of tissue inhibitors of metalloproteinases 2 and 3 (TIMP-2 and TIMP-3) in chondrocytes, thereby weakening cartilage resistance to matrix degradation and promoting KOA progression. Collectively, these studies indicate that exosomal miRNAs released by osteoclasts are associated with the promotion of KOA progression. Notably, one study found that RAB27A, a member of the RAS oncogene family, reduces osteoclast secretion of exosomal miRNAs by inhibiting key miRNA-processing enzymes or small interfering RNAs, thereby slowing KOA progression (134).

Platelet-rich plasma (PRP), which is rich in growth factors such as transforming growth factor-beta and vascular endothelial growth factor, has demonstrated therapeutic potential for alleviating KOA pain and improving joint function (135). One study found that aberrant expression of mitogen-activated protein kinase kinase kinase kinase 2 (MAP4K2) in KOA promotes phosphorylation, leading to activation of the Hippo pathway and inhibition of tumor necrosis factor alpha-induced protein 3 (TNFAIP3), resulting in NLRP3 inflammasome activation and exacerbation of the inflammatory response in KOA. Platelet-rich plasma-derived exosomal miR-25-3p was shown to target and inhibit MAP4K2, subsequently suppressing Hippo pathway activation and ultimately activating the anti-inflammatory protein TNFAIP3,thereby effectively inhibiting the inflammatory response in chondrocytes (132). This provides a promising approach for the treatment of KOA using platelet-derived exosomal miRNAs.

Furthermore, excessive free radicals in the joint can induce oxidative stress, posing a risk to subchondral bone matrix degradation and the progression of osteoarthritis (136). Because the subchondral bone of the joint is in close proximity to the cartilage, it plays a key role in cartilage repair research (137). Subsequent studies have found that human urine-derived stem cell exosomal miR-140-5p stimulates chondrocytes to inhibit oxidative stress responses, thereby reducing chondrocyte apoptosis, improving subchondral bone remodeling, and exerting protective effects on cartilage (131), as illustrated in Figure 2 and summarized in Table 1.

**Figure 2:**
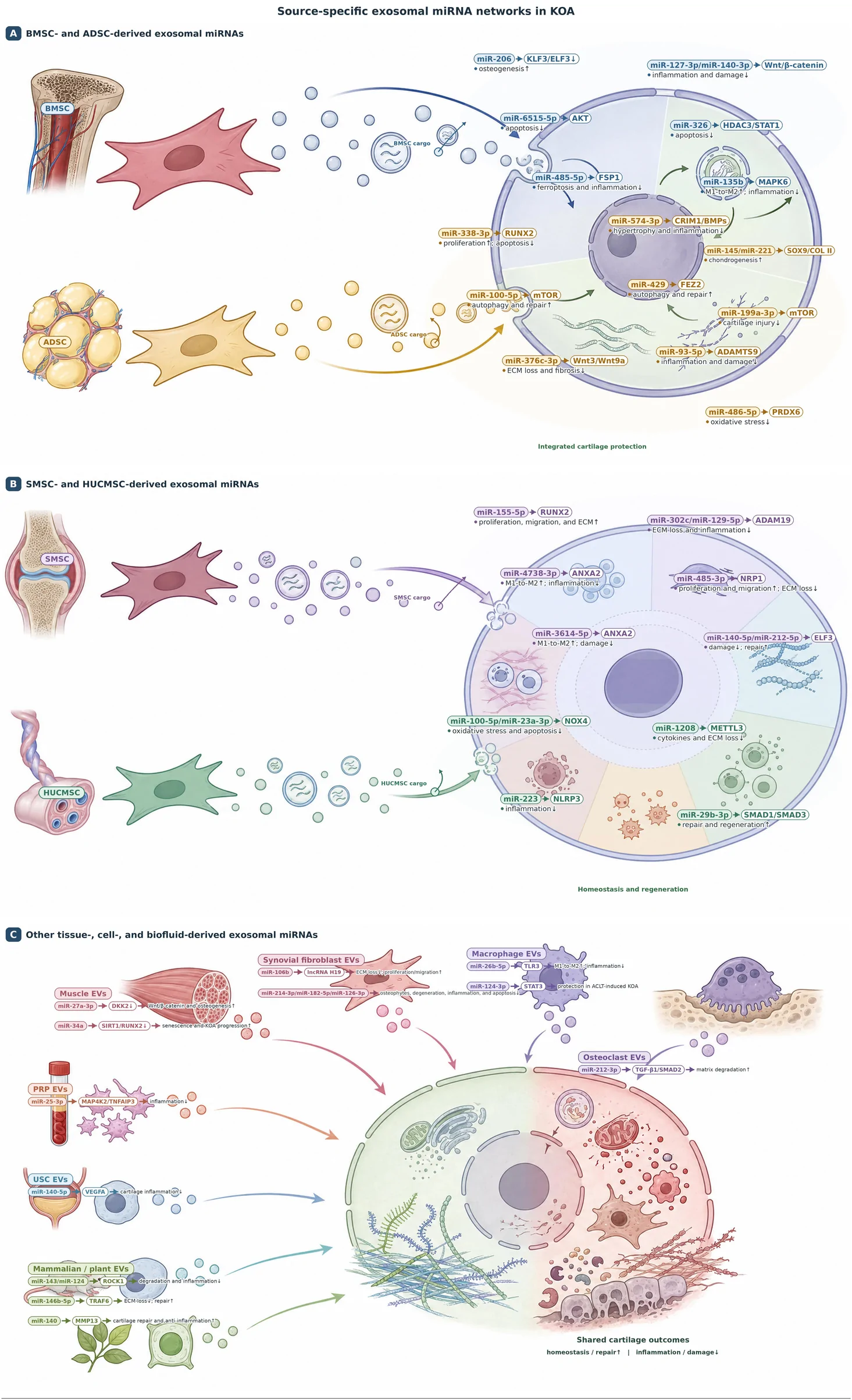
Schematic overview of the regulation of the articular cartilage microenvironment by exosomal miRNAs from distinct cellular sources. The figure depicts the molecular mechanisms through which exosomes exert biological effects on chondrocytes in the context of KOA pathogenesis and cartilage repair: **(A)** BMSC- and ADSC-derived exosomal miRNAs; **(B)** SMSC- and HUCMSC-derived exosomal miRNAs; **(C)** Other tissue-, cell-, and biofluid-derived exosomal miRNAs.

## 5 Discussion

Although exosomal miRNAs derived from diverse cellular origins demonstrate considerable therapeutic promise for knee osteoarthritis (KOA), the vast majority of existing evidence remains confined to the preclinical stage. Mesenchymal stem cell (MSC)-derived exosomal miRNAs, particularly those from bone marrow MSCs (BMSCs) and human umbilical cord MSCs (HucMSCs), have been shown to operate via multifaceted mechanisms, primarily involving Wnt/β-catenin signaling modulation, ferroptosis inhibition, and macrophage polarization. However, their clinical translation is hindered by substantial hurdles, including standardization of *ex vivo* biomanufacturing, batch-to-batch heterogeneity, and potential allogeneic immunogenicity; adipose-derived stem cell (ADSC) exosomes face comparable translational bottlenecks. Furthermore, exosomal miRNAs from synovial fibroblasts and macrophages display a dual character—either exacerbating cartilage degradation or facilitating repair—underscoring the necessity for precise source control and rigorous phenotypic validation. In contrast, skeletal muscle-derived exosomal miRNAs possess distinctive advantages: they can be induced non-invasively through physical exercise, thereby circumventing the immunological risks associated with allogeneic cell sources. Crucially, exercise dynamically remodels the muscle exosomal miRNA landscape, shifting it from a pro-senescent, pro-inflammatory profile (such as elevated miR-34a levels under atrophy) toward an anti-aging, osteogenic phenotype (marked by miR-27a-3p upregulation), thereby offering a biologically rational and clinically plausible non-invasive strategy for KOA management. However, it must be acknowledged that current evidence directly investigating skeletal muscle-derived exosomal miRNAs in patients with KOA remains limited; future research should explore the direct mechanistic pathways linking exercise-regulated muscle-derived miRNAs to knee osteoarthritis. A comparative overview of the advantages and limitations of exosomes from different cellular sources is provided in Table 2.

**Table 2:** Comparative analysis of advantages and limitations of exosomal miRNA sources in the context of knee osteoarthritis.

Cell Source	Advantages	Limitations
BMSCs	Multilineage differentiation potential; robust paracrine activity; strong preclinical evidence across Wnt/β-catenin, PI3 K/AKT/mTOR, ferroptosis, and macrophage polarization axes.	Invasive collection procedure; risk of donor-site morbidity; allogeneic immunogenicity; substantial batch-to-batch variability; high *ex vivo* manufacturing costs; reduced therapeutic potency in aged or inflamed donor microenvironments; no direct clinical evidence in KOA
ADSCs	Highly accessible tissue source; superior chondrogenic capacity relative to other MSC subtypes; greater tolerance to inflammatory stimuli; diverse miRNA cargo targeting autophagy, Wnt/β-catenin, and oxidative stress pathways.	Allogeneic immunogenicity; *ex vivo* production standardization challenges; dependence on intra-articular delivery; no direct clinical evidence in KOA; variable yield depending on anatomical depot (IPFP vs. subcutaneous)
SMSCs	Proximity to joint pathology; intrinsic chondrogenic differentiation capacity; relevant miRNA cargo (miR-155-5p, miR-485-3p, miR-3614-5p) with demonstrated effects on chondrocyte proliferation and macrophage polarization.	Highly invasive collection requiring arthroscopy; limited donor availability; reduced cell yield; incompletely characterized mechanistic profiles; no clinical evidence in KOA; potential for pathological phenotypic variation in OA synovium
HucMSCs	Non-invasive, ethically uncontroversial collection; strong osteogenic and angiogenic capacity; miRNA cargo (miR-100-5p, miR-1208, miR-29b-3p) targeting oxidative stress, inflammation, and cartilage repair; only source with preliminary first-in-human clinical safety data in KOA	Allogeneic origin with potential immunogenicity; standardization of clinical-grade manufacturing remains challenging; single-arm, uncontrolled clinical trial limits efficacy conclusions; insufficient long-term follow-up data; therapeutic effects attributable to specific miRNAs not yet isolated
Skeletal muscle-derived	Uniquely non-invasive induction via exercise; autologous origin minimizes immunogenic risk; dynamically reprogrammable miRNA cargo (miR-34a, miR-27a-3p) in response to exercise type, intensity, and duration; systemic circulatory delivery enables multi-joint reach; no *ex vivo* manufacturing required	no KOA-specific clinical studies; muscle-derived EVs constitute only ∼1%–5% of total circulating EV pool; no validated muscle-specific surface markers for precise attribution; optimal exercise prescription for therapeutic miRNA remodeling undefined; direct effects on articular cartilage not yet established
Synovial fibroblast-derived	Inherently joint-proximal source; some miRNAs (miR-214-3p, miR-182-5p, miR-126-3p) demonstrate cartilage-protective and anti-inflammatory effects; mechanistically relevant to local joint pathology	Predominantly pathological in the OA context; several miRNAs (miR-19b-3p, miR-106b, miR-142-5p) actively exacerbate chondrocyte ferroptosis and cartilage injury; requires precise phenotypic control; invasive collection; no clinical evidence; dual nature limits therapeutic predictability
Macrophage-derived	M2 macrophage-derived miRNAs (miR-26b-5p, miR-124-3p) exert dual chondroprotective and anti-inflammatory effects; mechanistically relevant to synovial immune modulation in KOA	M1 macrophage-derived miRNAs (miR-363) exacerbate cartilage injury; phenotypic stability of polarized macrophages difficult to maintain *ex vivo*; complex isolation procedures; no standardized therapeutic protocols; no clinical evidence in KOA
Osteoclast-derived	Mechanistically relevant to subchondral bone–cartilage crosstalk; inhibiting specific miRNAs (miR-212-3p) may represent a therapeutic target for cartilage repair	Predominantly disease-promoting in KOA context; most characterized miRNAs (miR-212-3p, miR-21a-5p, miR-214-3p, miR-30) accelerate cartilage matrix degradation; complex isolation; no clinical evidence; limited therapeutic applicability without precise targeting
Platelet-rich plasma (PRP)-derived	Minimally invasive autologous collection; rich in growth factors; miR-25-3p demonstrated anti-inflammatory chondroprotective effects via MAP4K2/Hippo/TNFAIP3 axis; established clinical use of PRP in KOA provides regulatory precedent	Exosomal miRNA cargo not yet fully characterized; evidence limited to single miRNA study; PRP composition highly variable across preparation protocols; therapeutic effects difficult to attribute specifically to exosomal miRNAs vs. growth factors; limited controlled clinical evidence for exosomal miRNA component
Human urine-derived stem cells	Completely non-invasive collection; miR-140-5p demonstrated cartilage-protective effects via VEGFA suppression; reduced oxidative stress and improved subchondral bone remodeling in preclinical models	Highly limited evidence base (single miRNA study); collection and isolation protocols not standardized; therapeutic potential largely unexplored; no clinical evidence in KOA; cell characterization and exosome yield consistency unclear
Mammalian and plant-derived	Novel and diverse bioactive cargo; natural anti-inflammatory and antioxidant properties; miR-140, miR-143, miR-124, miR-146b-5p demonstrated cartilage repair and inflammation inhibition in KOA models; potential for cost-effective large-scale production	Mechanistic basis poorly characterized; cross-species miRNA functional validity uncertain; no standardized isolation or quality control protocols; no clinical evidence; cargo composition varies significantly by source organism and extraction method

KOA, knee osteoarthritis; BMSCs, bone marrow mesenchymal stem cells; ADSCs, adipose-derived stem cells; SMSCs, synovial mesenchymal stem cells; HucMSCs, human umbilical cord mesenchymal stem cells; IPFP, infrapatellar fat pad; EV, extracellular vesicle; PRP, platelet-rich plasma; OA, osteoarthritis; MSC, mesenchymal stem cell.

At present, the clinical translation of exosomal miRNA-based therapeutics across all cellular sources is still in its infancy. Notably, clinical-grade extracellular vesicles derived from HucMSCs have been standardized and subjected to intra-articular validation (138–140). Characterization of their exosomal miRNA and protein profiles has further confirmed the feasibility of standardized vesicle manufacturing for clinical applications. The efficacy and biosafety of HucMSC-Exos were initially validated in *in vitro* cellular assays and *in vivo* animal models. Subsequently, twelve patients with moderate KOA received a single intra-articular injection of HucMSC-Exos and completed a 12-month follow-up (141). No adverse events were recorded during the follow-up period, and patients achieved significant, sustained improvements in both pain relief and functional mobility. Imaging evaluations confirmed that intra-articular HucMSC-Exos did not induce joint structural damage or accelerate cartilage degeneration. This clinical study provides preliminary evidence supporting the clinical safety of exosome-based therapy for KOA, offering valuable insights for future trials in this field. However, critical limitations of this study must be explicitly acknowledged: the trial enrolled only 12 patients and lacked a placebo or active control arm, precluding definitive conclusions regarding therapeutic efficacy. Moreover, while the 12-month follow-up provided valuable short-term safety data, it remains insufficient to evaluate the long-term durability of the observed clinical benefits.

Notably, the vast majority of mechanistic insights to date have been derived from IL-1β-stimulated *in vitro* chondrocyte cultures or surgically induced animal models, which fail to fully recapitulate the complex, multifactorial, and chronic pathophysiological progression of human KOA. Furthermore, the lack of standardized exosome isolation and characterization protocols across laboratories introduces substantial methodological heterogeneity, limiting cross-study comparability. Specifically regarding muscle-derived exosomal miRNAs, the optimal exercise prescription—including exercise modality, intensity, duration, and frequency—capable of inducing therapeutically meaningful miRNA remodeling in KOA patients has yet to be defined. Beyond these methodological concerns, several intrinsic limitations of this review warrant explicit acknowledgment. As a narrative rather than systematic review, the literature synthesis is inherently susceptible to selection bias, and the possibility of relevant studies being overlooked cannot be excluded. Moreover, determining the precise tissue origin of circulating extracellular vesicles remains methodologically challenging, as no validated muscle-specific surface markers currently exist, limiting the confidence with which circulating miRNA changes can be attributed to skeletal muscle specifically. Additionally, direct head-to-head comparative studies evaluating the relative therapeutic efficacy of exosomes from different cellular sources within the same KOA model are currently absent, precluding evidence-based prioritization of sources for clinical translation. A significant strength of this review lies in its adoption of the muscle–joint axis framework, systematically comparing muscle-derived exosomal miRNAs with other well-characterized cell sources to provide a structured roadmap for future research and clinical translation. However, current evidence is heavily skewed toward preclinical models, and the scarcity of human clinical data significantly constrains the translational reliability of current conclusions. Future investigations should prioritize the development of muscle-specific extracellular vesicle tracking tools, the design of clinical exercise trials combining quantitative joint imaging with circulating exosome profiling, and the execution of adequately powered, randomized controlled trials to definitively establish the clinical efficacy and long-term safety of exosomal miRNA-based interventions in KOA.

## 6 Conclusion

Because articular cartilage degradation is a central pathological hallmark of knee osteoarthritis (KOA) and chondrocytes are the principal resident cells within cartilage, this review focused on the regulatory effects of exosomal microRNAs (miRNAs) from diverse cellular origins on chondrocyte biology. Accumulating preclinical evidence suggests that exosomal miRNAs derived from bone marrow, adipose tissue, synovium, umbilical cord, synovial fibroblasts, and macrophages may modulate key pathological processes implicated in KOA. Among these, skeletal muscle-derived exosomal miRNAs may possess distinctive biological features and potential therapeutic relevance. Targeted exercise interventions that modulate atrophy- and senescence-associated exosomal miRNAs may represent a promising avenue for further investigation in KOA. Current preclinical evidence suggests that physical exercise may mediate molecular communication between skeletal muscle and the joint through an exosomal miRNA signaling axis. Although mechanistic studies provide evidence supporting this muscle–joint communication axis, the available evidence remains predominantly preclinical, with direct validation in humans still lacking. Therefore, the exosomal miRNA signaling axis should currently be considered a promising, mechanistically supported hypothesis rather than an established therapeutic mechanism.

From a clinical translation perspective, preliminary human studies of extracellular vesicle-based interventions in KOA have reported acceptable safety and joint tolerability; however, their therapeutic efficacy remains to be established in adequately powered randomized controlled trials. For muscle-derived exosomal miRNAs specifically, direct clinical validation in humans has not yet been established. Future research should prioritize the development of standardized isolation and characterization protocols for muscle-specific extracellular vesicles, the design of clinical exercise trials integrating quantitative joint imaging with circulating exosome profiling, and rigorous head-to-head comparative evaluations across distinct exosome sources.

In summary, muscle-derived exosomal miRNAs may have distinctive translational potential because of their responsiveness to exercise and their possible role in systemic muscle–joint communication. Nonetheless, current mechanistic evidence derives predominantly from reductionist *in vitro* and murine models, whose translational fidelity to human KOA remains uncertain. Human clinical evidence supporting exosomal miRNA-based interventions remains limited, and their long-term safety and therapeutic durability ultimately require confirmation through adequately powered, rigorously designed clinical trials.
